# Arsenic on Children’s Hands: Le et al. Respond

**Published:** 2005-08

**Authors:** X. Chris Le, Elena Kwon, Hongquan Zhang, Zhongwen Wang, Gian S. Jhangri, Xiufen Lu, Xing-Fang Li, Nelson Fok, Stephan Gabos

**Affiliations:** University of Alberta Edmonton, Alberta, Canada, E-mail: xc.le@ualberta.ca; Capital Health Edmonton, Alberta, Canada; Alberta Health and Wellness Edmonton, Alberta, Canada

We appreciate the comments of Zagury and Pouschat and their support of our overall conclusions presented in our article (Kwon et al. 2004). In response to their thoughtful comments, we would like to offer the following clarifications.

In the introduction of our article (Kwon et al. 2004), we cited Balasoiu et al. (2001), Zagury et al. (2003), and others (Stilwell and Gorny 1997; Townsend et al. 2003), who examined arsenic in soil and sand samples from the field or from the laboratory. These references provide the readers with useful background information on the sources and levels of potential arsenic exposure. Examining the distribution, partitioning, and concentration of arsenic in the environmental media (e.g., soil, sand, water, and wood surface) appeared to be the primary objectives of these studies. Arsenic levels had not been directly measured on the hands of children after contact with either chromated copper arsenate (CCA)-treated wood or soil in playgrounds until our study (Kwon et al. 2004).

Because the primary objective of our study was to determine the amount of arsenic on the hands of children after playing in playgrounds, we did not focus on the characterization of arsenic in the soil. Although we determined the levels of arsenic in the composite soil samples from the playgrounds, a detailed characterization of the spatial distribution of arsenic was outside the scope of our study. We agree that the concentration of arsenic in the soil samples varies greatly with the sampling protocols and the location of the samples with respect to the CCA-treated wood structures (Chirenje et al. 2003; Stilwell and Gorny 1997; Zagury et al. 2003; Ursitti et al. 2004). Our composite soil samples could not provide any information on the spatial distribution of arsenic concentration in soil samples collected from the playgrounds. These composite samples were obtained from areas under decks and away from any wood structures. We did not collect soil/sand samples from areas immediately adjacent to the CCA-treated wood. Further studies to understand the distribution of arsenic in playgrounds would benefit from extensive collection and analysis of soil samples from different locations in the playgrounds.

We clearly stated that “children playing in playgrounds constructed with CCA-treated wood have approximately five times more arsenic on their hands than do those playing in playgrounds that do not have CCA-treated wood structures.” We also feel that “it is important to point out to the general public that arsenic is naturally present in the soil and sand regardless whether the playgrounds contain CCA-treated wood structures.” During our study, we found that many of the parents of the participating children did not know that arsenic was naturally present in the environment, albeit with varying concentrations. They thought that if there was any arsenic, it must have been added to the environment by someone. Conversely, if there was no added “synthetic” arsenic, they did not consider arsenic as a potential health concern. This attitude toward toxic substances (natural versus synthetic) can be counterproductive in the effort to achieve the goal of protecting public health. Properly informing the public that arsenic is naturally present in the soil helps people to understand that it is important for children to wash their hands after playing, regardless of whether the playgrounds contain CCA-treated wood structures. The hand–mouth activities of young children can result in the ingestion of arsenic that may be adsorbed on their hands. Children should wash their hands after playing to reduce their potential exposure to arsenic.

We agree with Zagury and Pouschat that “potential ingestion of arsenic from soil under CCA-treated structures should not be neglected.” All efforts need to be made to minimize children’s exposure to the toxic species of arsenic.
